# Which young physicians are satisfied with their work? A prospective nationwide study in Norway

**DOI:** 10.1186/1472-6920-5-19

**Published:** 2005-06-02

**Authors:** Kristine Benedictow Finset, Tore Gude, Erlend Hem, Reidar Tyssen, Oivind Ekeberg, Per Vaglum

**Affiliations:** 1 Department of Behavioural Sciences in Medicine, Institute of Basic Medical Sciences, Medicine, University of Oslo, Postbox 1111 Blindern, NO-0317 Oslo, Norway

## Abstract

**Background:**

Few studies have investigated personality and medical school variables in regard to job satisfaction after graduation. It is of great importance to investigate these factors because this information may be used in the recruitment/admittance process to medical schools, and possibly to improve medical education.

**Methods:**

We conducted a nationwide prospective 10-year follow-up study of medical students at all medical schools in Norway. They were approached three times during their medical training: at very beginning (T1), in the middle (T2), in the last year of medical school (T3), and then four years after graduation (T4). There were 210 participants who responded on all four occasions. Job satisfaction was measured with the Job Satisfaction Scale, which was used as the outcome variable. In addition to conducting multiple regression analysis for the total sample, we also conducted similar analyses separately for men and women.

**Results:**

Among the demographic and personality variables, 'having a father who is a physician' and 'interpersonal functioning (being withdrawn)' were significantly associated with job satisfaction at T4. Among the medical school variables, 'well-being with peers', 'identification with the doctor's role at the end of curriculum', 'perceived medical school stress', and 'perceived clinical skills' were significantly associated with job satisfaction. In the multiple regression analysis only 'father as a physician' and 'perceived clinical skills' yielded an independent influence on the outcome variable in separate analyses within sub-groups of male and female students, 'perceived clinical skills' differentiated among woman only, while 'well-being with peers' differentiated only among men.

**Conclusion:**

The main finding of this study is that the young physicians who are the most satisfied in their work are those whose fathers are physicians and those who have a high level of perceived clinical skills at the end of medical school. There are also differences in regard to predictors of job satisfaction among men and women. These findings indicate that medical schools should invest substantial effort in clinical skills training, and this seems to be especially important among female students.

## Background

Physicians' job satisfaction is important because it may affect patient satisfaction [1] and patient adherence [2] and may be inversely associated with level of stress as well as burnout [3,4]. Most studies have emphasized the importance of several aspects of the current work situation that contribute to the job satisfaction of physicians [5]. Both sense of autonomy [6] and adequate time with patients [7] have been shown to be of particular importance. The current work situation will possibly explain most of the variations in job satisfaction among young physicians. On the other hand some researchers found that differences in current work situation for pre-registered house officers did not predict whether or not they were rated as good or bad [8], on this basis the researchers propose that there are personality characteristics by the doctors themselves that make the difference. Nevertheless, are there also other factors that are important determinants of subsequent job satisfaction that could be identified during the time spent at medical school? Which medical students will truly be satisfied working as physicians? If we could identify the characteristics of such students, we might have information that could be used in the recruitment/admittance process of medical schools, and could also possibly improve our medical education. One study has reported a relationship between learning style in medical school and approach to work several years later [3]. To our knowledge, however, there are few other prospective studies that have addressed these important questions. Therefore, we find it of great value to further investigate these aspects of physician job satisfaction.

There are several factors that could be possible predictors of young physicians' job satisfaction. Age and sex may be important. Previous cross-sectional studies have, however, reported conflicting results [9-11]. Coming from a family of physicians may imply a high motivation and realistic expectations regarding working as a physician. In a previous study from this longitudinal project, having a father who was a physician predicted level of ambition in medical students [12]. Regarding the importance of personality traits, one study found that extraversion had a positive effect and neuroticism a negative effect on job satisfaction [13]. This is not surprising, given that extroversion and neuroticism are well known to affect satisfaction and well-being in general [14]. Interpersonal functioning as a personality trait is important for mastering relationships with patients and colleagues, and could therefore influence later job satisfaction. In addition, self esteem and the tendency to react with nervous symptoms could also be of importance.

Concerning medical school variables, one would expect that students who later will be satisfied with their work as physicians have reached a higher level of identification with the doctor's role [15], and have attained a high level of confidence in their own clinical skills by the end of medical school. In a review article on the performance-satisfaction relationship, Judge, Thoresen, Bono & Patton [16] propose the possibility that performance precede satisfaction. It is therefore of interest to further investigate this relationship.

Students with a high level of perceived medical school stress and/or those who are interacting poorly with student colleagues might be at risk of experiencing the same difficulties when they are working as physicians.

Based on these considerations, we have used data from a prospective longitudinal study of a nationwide sample of Norwegian medical students that were followed from the first month of university, examined again after three years, again at the end of the curriculum and finally four years later, to identify predictors of job satisfaction after graduation.

We hypothesized that the following variables would influence job satisfaction four years after the end of medical school:

1) Demographic factors: sex, age and father being a physician

2) Personality factors and nervous distress: personality traits, self esteem interpersonal problems and nervous symptoms.

3) Medical school factors: type of medical school, perceived clinical skills, well being with peers, perceived medical school stress and identification with the doctor's role at the end of the curriculum.

## Methods

### Sample

Students entering medical school at the four Norwegian universities in 1993 (n = 421) were invited to participate. Data were collected in the first month of medical school (T1), in the middle of the third year (T2), at the end of the sixth year (T3) and four years after graduating (T4). The response rates at the four different collections are shown in table 1. At T2, not all of the students whom were invited to participate at T1 were available, most obviously due to drop-out from school. Therefore, the population of students varied from the one assessment time to the next, making it difficult to estimate the real response rate. All the way, those responding on all four occasions was 210 individuals (50% of the original population with N = 421), 59% of whom were women; this constituted the sample to be investigated. Thus, we would estimate the "true" response rate to be closer to 60% than to 50%.

**Table 1:** Respons rate at the four different occasions

Point of time	Response rate	Total response rate in percent	Response rate of original sample
1993 – T1	374	88%	-
1996 – T2	287	68%	77%
1999 – T3	238	56%	64%
2003 – T4	210	50%	56%

Mean age at T1 was 21.6 years (SD 2.6) with no significant sex differences. Students with foreign citizenship accounted for 2.5% of the sample. Education of the parents at college level or higher was 77% for the fathers and 74% for the mothers, and 14.8% (n = 31) of the sample had a mother and/or a father who was a physician. There were no participants who only had mother as a physician; either both parents or father only had this occupation. There were no significant differences between the response and non-response group at T4 regarding age or citizenship. The non-response rate was somewhat higher for men than for women (57% vs. 40%, Chi square = 10.07, p = .002).

There were no substantial differences between the four universities regarding the proportion of responders at T3 and responders at T4. Further descriptions of the sample and characteristics of the non-responders are accounted for else where [15].

The study was conducted in collaboration with the Norwegian Medical Association and with the approval of the National Data Inspectorate.

### Procedures

Data from all four universities were collected by mail and were anonymous, except for a code number (known only by the National Bureau of Statistics) linking data from all the four occasions to the same person.

### Outcome variable

Job satisfaction was assessed at T4 using a 10-item version of the Job Satisfaction Scale [17], which employed a seven-point Likert scale ranging from 1 = 'very satisfied' to 7 = 'very dissatisfied'. The validity and reliability of the scale has been found satisfactory [17]. Some examples of the items are: [how satisfied are you with...] 'the amount of responsibility you are given', 'your fellow workers', 'your hours of work' and 'your opportunity to use your ability'. For the sake of convenience, we inverted the scale so that a high score indicated high satisfaction. A Principal Component Analysis of the 10 items yielded a typical one-factor solution; therefore, the index score from all items was used as the outcome variable. The Kaiser-Meyer-Olkin value was satisfactory (.8) and the Bartlett's Test of Sphericity reached statistical significance. The eigenvalue of this component was: 3.945. Cronbach's α for the whole inventory was .80. The overall mean of Job Satisfaction in the sample was 5.18 (SD = .78). The median was 5.2.

### Predictors – bivariate analysis

All predictors are shown in Table 2, with mean, standard deviation (SD), reliability coefficient, and assessment time displayed. Co-linearity-analyses has been conducted for all the included predictors, values did not exceed .40.

**Table 2:** Mean, SD and assessment time.

	**Variable**	**Time**	**Mean (SD) and percent**
**Demographic variables**	Gender	T1	Women 59 % **Men **41%
	Age	T1	Mean = 21.6 (SD = 2.58)
	Father as physician	T1	No – 75.7% (n = 159) Yes – 14.8% (n = 31)

**Personality variables**	Basic Character Inventory	T1	**Vulnerability**: M = 3.8 (SD = 2.22) **Intensity: **M = 5.2 (SD = 2.22) **Control: **M = 3.2 (SD = 2.12) **Reality weakness**: M = 1.8 (SD = 1.67)
	Inventory of Interpersonal Problems	T2	**Submissiveness**: M = 0.7 (SD = 0.38) **Aggression**: M = 0.6 (SD = 0.37) **Sociability/Withdrawn**: M = 0.8 (SD = 0.43)
	Symptom checklist	T1	Mean = 0.55 (SD = 0.62)
	Self esteem	T1	Mean = 2.9 (SD = 0.62)

**Medical school variables**	Perceived Clinical Skills	T3	Mean = 5.0 (SD = 0.63)
	Well-being with peers	T2	Mean = 5.3 (SD = 1.00)
	Perceived medical school stress	T3	Mean = 2.43 (SD = 0.51)
	Identification with the doctor's role	T3	Mean = 4.9 (SD = 1.06)

**Outcome Variable**	**Job satisfaction**	T4	Mean = 5.2 (SD = .78)

T1, 1993 (beginning of medical school); T2, 1996; T3, 1999 (end of medical school); T4, 2003 (four years after graduation)

### Demographic variables

Sex (female = 1/male = 2), age, and the parents' occupations, specifically whether or not the parents were physicians, were recorded.

### Personality variables

Personality traits were measured using the 'Basic Character Inventory' [18,19], a 36-item questionnaire with sub-scales of vulnerability (neuroticism), intensity (extroversion), control, and reality weakness, requiring yes/no answers. The data were collected at T1.

One instrument assessed nervous symptoms: the Symptom Checklist-5 [20] with a five-point scale ranging from 0 = 'no problem' to 4 = 'severe problems'. The data were collected at all assessments, but data from T1 were used in this study.

One instrument measured interpersonal functioning: the Inventory of Interpersonal Problems (IIP) (64 items) [21], a five-point scale ranging from 0 = 'no problem' to 4 = 'severe problems'. The data were collected at T2. The 64 items of this inventory were subjected to Principal Components Analysis (PCA). Prior to performing PCA, the suitability of data for factor analysis was assessed. The Kaiser-Meyer-Olkin value was satisfactory (.8) and the Bartlett's Test of Sphericity reached statistical significance, supporting the factorability of the items.

The scree-plot indicated that a three-component solution best fitted the data. The eigenvalues of these three components were: 12.356, 5.180 and 3.960. Together these three components explained a total of 33.6% of the variance. To facilitate the interpretation of these three components, a Varimax rotation was performed. Items with sufficient factor loadings on the first component only constituted an index called 'Submissiveness', consisting of items such as 'It is hard for me to be self-assertive', 'It is hard for me to set limits' and 'It is hard for me to show anger'. Items with sufficient factor loadings on the second component only constituted an index called 'Sociability/Withdrawn', consisting of items such as 'I keep people too much at a distance', 'It is hard for me to trust other people' and 'It is hard for me to really care about another person's problems'. Items with sufficient factor loadings on the third component only constituted an index called 'Aggression' and consisted of items such as 'I lose my temper too much', 'I argue too much' and 'I manipulate others too much'.

Self-esteem is a measurement derived from the vulnerability dimension of the original BCI-136 item version [18,22]. The variable used in the analyses was an index computed from eight items with a four-point scale at T1 ranging from 1 = 'do not agree' to 4 = 'agree'. A typical item was: "I feel most often that others perform better than I do myself".

### Medical-school factors (collected at T3)

The curriculum differed in some respects between the four Norwegian medical schools. When the students started their medical education, two sites (Oslo and Bergen) organized their curriculum according to the traditional division between pre-clinical and clinical parts, with a comprehensive exam in between. At the two other sites (Trondheim and Tromsø), the teaching of pre-clinical and clinical subjects was integrated, with no main exam completed prior to data collection at T2. For the multivariate analyses, the four study sites were computed as dummy variables, with one of the integrated schools as the reference value.

Two questions further measured the students' well-being with peers, 'To what degree do you feel secure among your fellow students' and 'To what degree do you feel satisfied with your fellow students'. Both items were measured on a seven-point Likert scale, ranging from 1 = 'to a very little degree' to 7 = 'to a very large degree'.

Perceived medical-school stress (PMSS) was measured at T3 using an instrument developed by Vitaliano et al. [23] and modified by Bramness et al. [24], which has previously shown good reliability and validity. The instrument consists of thirteen items with a five-point scale ranging from 1 = 'low stress' to 5 = 'high stress'.

Identification with the doctor's role at the end of the curriculum was measured using four items, with a seven-point Likert scale ranging from 1 = 'never/little' to 7 = 'always/very much'; an example of the questions is 'I feel like a doctor in the emergency room'. The construction of this variable has been described elsewhere [15].

Self-assessed clinical skills were measured using 'Perceived Clinical Skills' (PCS) (in an earlier study called Perceived Recording Skills) at T3 with six items, of which three items covered confidence in own competence, which has been used and validated earlier [15]. A typical item covering confidence in clinical skills is: 'I feel confident about examinations to be done', using a response scale from 1 = 'never' to 5 = 'always'. For an overview of the measures and instruments, see Table 2.

### Multivariate analysis

Block-wise multiple regression analyses were performed, using job satisfaction scores as the dependent variable. The predictors were entered in three blocks: demographics ('sex', 'age' and 'father as physician') were entered in the first block, the only bivariate significant personality variable ('interpersonal problems – withdrawn') was entered in a separate block, and medical school variables ('well-being with peers', 'perceived medical school stress', 'perceived clinical skills' and 'identification with the doctor's role') were entered in the third and final block.

We also conducted identical multiple regression analyses separately for men and women.

### Statistics

Means, median, correlations and block-wise multivariate analyses were conducted to investigate the relationship between the independent variables and job satisfaction. A p value < .05 was used as the level of significance. SPSS version 12.0 was used for statistical analyses.

## Results

Bivariate correlations between predictors and job satisfaction, and alpha-levels are shown in Table 3. One of the demographic and one of the personality variables were significantly related to job satisfaction four years after graduation: father being a physician (r = .161, p = .028) and the interpersonal index 'withdrawn' (from IIP) (r = -.255, p < .001). Among the medical school variables, students' well-being with peers (r = .221, p = .001), identification with the doctor's role (r = .240, p = .001), perceived medical school stress (r = -.236, p = .001), and perceived clinical skills (r = .240, p < .001) were significantly correlated with job satisfaction.

**Table 3:** Bivariate correlations between predictors and job satisfaction.

**Predictors**	**Job Satisfaction**	
**Background variables**		
Gender	-.026	
Age	.019	
Study location	ns.	
Parent as physician (father)	. 161 * (p = .028)	
**Personality variables**		
BCI – Vulnerability	-.101	Cronbach's Alpha:.691
BCI – Intensity	.050	Cronbach's Alpha: .681
BCI – Control	-.036	Cronbach's Alpha: .663
BCI – Reality Weakness	.031	Cronbach's Alpha: 59.4
Symptom distress (SCL-5)	-.022	Cronbach's Alpha: .802
Interpersonal – dominating	-.072	Cronbach's Alpha: .817
Interpersonal – submissive	-.110	Cronbach's Alpha: .848
Interpersonal – withdrawn	-.255 *** (p < .001)	Cronbach's Alpha: .764
Self-esteem (T1)	.093	Cronbach's Alpha:..852
**Medical school variables**		
Well-being with peers	.221** (p = .001)	Jus two items.
Identification with doctor's role	.240 ** (p = .001)	Cronbach's Alpha: .833
Perceived medical school stress	-.236 ** (p = .001)	Cronbach's Alpha: .782
Perceived clinical skills	.287 *** (p < .001)	Cronbach's Alpha: .563

* p < .05 ** p < .01 *** p < .001

### Multivariate analyses

The predictors that had a significant bivariate correlation with job satisfaction were entered blockwise in a linear multiple regression analysis, with job satisfaction as the dependent variable (Table 4). In the final model, two predictors independently explained variance in job satisfaction: one was whether father was a physician, the other was level of perceived clinical skills, which together explained a total of 13.9% (R^2 ^.139) of the variance. The change in adjusted R^2 ^was significant from step one to two (F = 12.74, p < .001) and from step two to three (F = 4.63, p = .001).

**Table 4:** Multivariate analysis of bivariate correlating predictors with job satisfaction

	**Block 1**	**Block 2**	**Block 3**
**Gender**	.018	.043	-.004
**Age**	.020	.019	-.001
**Father dr.**	.165*	.181*	.147*
**IIP-W.**		-.254***	-.113
**Well-b. peers**			.099
**PMSS**			-.112
**PCS**			.171*
**Ident**			.089
**Adj. R^2^**	.011	.070	.139

* p < .05 ** p < .01 *** p < .001

We finally conducted the same blockwise analyses in sub-groups of female and male students separately. In these analyses, a different pattern emerged. Among male physicians, only well-being with peers (stand. beta .28, p = .025) contributed significantly, explaining a total of 13.8% (R^2 ^.138) of the variance. 'Father being physician' did not contribute significantly in the male sub-sample (stand. beta .041, p = .728). Among female physicians, 'perceived clinical skills' was the only significant contributor (stand. beta .25, p = .013), explaining a total of 14.4% (R^2 ^.144) of the variance. 'Father being a physician' only reached borderline significance in the female sample (stand. beta .165, p = .076) (not shown in table).

## Discussion

The main finding of this study was that young physicians who were the most satisfied in their work were those that had a father who was a physician and those who had a high level of perceived clinical skills at the end of medical school. It may be argued that confidence in own (perceived) clinical skills is reflecting a general self-esteem as a personality trait rather than being linked to real clinical competence. In this study, however, we neither found a significant correlation between the self-esteem index nor the personality variables (BCI) and job satisfaction, indicating that confidence in clinical skills in this respect do measure more than personality characteristics. The impact of self-assessed clinical skills, most probably influencing the management of challenges in medical work, is understandable and consonant with results from other studies [15,19]. An optimal and balanced confidence in clinical skills are not only of utmost importance for the benefit of the patients, but also, as this study shows, significant for job satisfaction after graduation. This finding emphasizes the importance of medical school training of clinical skills that in turn will help promoting an optimal and balanced confidence. In addition, we found that interpersonal functioning is important for job satisfaction, but this factor is most probably goes through perceived clinical skills as this variable is the heaviest influencing one in the block of medical school variables. This might indicate that socially withdrawn students achieve less confidence by clinical training which may reduce their development of clinical competence.

'Father as physician' also contributed significantly to the level of job satisfaction in the final model, and is consonant with a similar influence upon ambition in medical students [12]. This family constellation may reduce the expectations of how satisfying the working situation for a physician should be. When each sex were analysed separately, the effect of 'father as physician' was most important among the female physicians. Being the daughter of a physician may imply a better role model when entering a still male dominant profession, and may also evoke more positive attitudes from colleagues. Although this constellation seems to be a valuable indication of job satisfaction, for many reasons it is still not useful as a criterion for admission to medical school.

When multiple regression analyses were conducted for men and women separately, other interesting differences also emerged. In relation to job satisfaction, perceived clinical skills differentiated women only, while well-being with peers differentiated only men. These findings were, to some degree, unexpected. It can be explained by the assumption that it is more important for female students' satisfaction with work to be competent in clinical skills and feel safe in their relationship with patients, while among men, the satisfaction depends more on their social skills in relating to colleagues. These differences should be further explored.

There are some limitations to this study. Even with the difficulties in assessing an objective non-response rate, there is no doubt that the size of the investigated sample is reduced to at least 60% compared to the student-cohort we intended to follow through the 10 years. As no sex differences in the level of job satisfaction were detected, the higher response rate among women should not bias our results. Although there was a limited response rate, the lack of differences in level of job satisfaction between sexes should not influence the representativity of the sample. Concerning the variable of the father being a physician, the N became small in the separate analyses of men and women, thereby increasing the risk of type II errors. In total, a variance of 14% was explained. This may initially seem small and insignificant. On the other hand, in this study we were mainly occupied with early predictors of job satisfaction, and this probably accounts for the low level of explained variance

## Conclusion

We have found that having a father as physician and the level of perceived clinical skills at the end of medical school were related to job satisfaction four years after graduation; this effect remained when controlling for age, sex and personality. Medical schools should therefore emphasize clinical skills training. This is especially important among female students.

## Competing Interests

The author(s) declare that they have no competing interests.

## Authors' Contributions

KBF acted as the principal investigator. TG and PV contributed substantially to the whole study. EH, RT and OE contributed to the interpretation of data and drafting of the manuscript.

## Pre-publication history

The pre-publication history for this paper can be accessed here:


